# IL-7 and SCF Levels Inversely Correlate with T Cell Reconstitution and Clinical Outcomes after Cord Blood Transplantation in Adults

**DOI:** 10.1371/journal.pone.0132564

**Published:** 2015-07-15

**Authors:** Ioannis Politikos, Haesook T. Kim, Sarah Nikiforow, Lequn Li, Julia Brown, Joseph H. Antin, Corey Cutler, Karen Ballen, Jerome Ritz, Vassiliki A. Boussiotis

**Affiliations:** 1 Department of Medicine and Division of Hematology Oncology, Beth Israel Deaconess Medical Center, Boston, MA, United States of America; 2 Department of Computational Biology, Dana-Farber Cancer Institute, Boston, MA, United States of America; 3 Department of Medical Oncology, Dana-Farber Cancer Institute, Boston, MA, United States of America; 4 Bone Marrow Transplantation Unit, Massachusetts General Hospital, Boston, MA, United States of America; Fujita Health University, School of Medicine., JAPAN

## Abstract

Recovery of thymopoiesis is critical for immune reconstitution after HSCT. IL-7 and SCF are two major thymotropic cytokines. We investigated whether the kinetics of circulating levels of these cytokines might provide insight into the prolonged immunodeficiency after double umbilical cord blood transplantation (dUCBT) in adults. We examined plasma levels of IL-7 and SCF, T-cell receptor rearrangement excision circle (TREC) levels and T cell subsets in 60 adult patients undergoing dUCBT. Median levels of IL-7 increased by more than 3-fold at 4 weeks and remained elevated through 100 days after dUCBT. SCF showed a less than 2-fold increase and more protracted elevation than IL-7. IL-7 levels inversely correlated with the reconstitution of various T cell subsets but not with TRECs. SCF levels inversely correlated with reconstitution of CD4^+^T cells, especially the naïve CD4^+^CD45RA^+^ subset, and with TRECs suggesting that SCF but not IL-7 had an effect on thymic regeneration. In Cox models, elevated levels of IL-7 and SCF were associated with higher non-relapse mortality (p = 0.03 and p = 0.01) and worse overall survival (p = 0.002 and p = 0.001). Elevated IL-7 but not SCF was also associated with development of GvHD (p = 0.03). Thus, IL-7 and SCF are elevated for a prolonged period after dUCBT and persistently high levels of these cytokines may correlate with worse clinical outcomes.

## Introduction

Umbilical cord blood (UCB) is a valuable alternative source of hematopoietic stem cells (HSC) for patients in need of allogeneic HSC transplantation (HSCT) who lack a suitable sibling or unrelated adult donor. The use of two UCB units has circumvented the HSC dose limit and has shortened the time to engraftment in adults [1, 2]. However, despite the improved myeloid engraftment, lymphoid reconstitution after UCB transplantation (UCBT) is often delayed, even with the use of two UCB grafts [3–5].

Patients undergoing HSCT develop a period of profound lymphopenia following conditioning, regardless of the graft source. The quantitative and qualitative T cell reconstitution after HSCT depends on two different pathways [6]. The thymic-independent pathway predominates in the early posttransplant period and is characterized by the homeostatic peripheral expansion of adoptively transferred donor T cells contained in the graft, or conditioning resistant recipient T cells. Homeostatic peripheral expansion of T cells is mediated by elevated levels of lymphocyte homeostatic cytokines, among which IL-7 has been identified to play a major role [7]. However, restoration of an immunologically competent T cell compartment with broad antigenic specificity eventually depends on the de novo production of naïve T cells by the thymus [8, 9]. This thymus-dependent pathway of T cell restoration is a prolonged process and involves the migration and thymus seeding of lymphocyte progenitors that arise from the engrafted, donor-derived, HSCs. These lymphocyte progenitors subsequently undergo intrathymic T cell proliferation, differentiation and selection, under the influence of cytokines and surface proteins provided by thymic epithelial cells. The small number of T cells that successfully complete the thymus dependent pathway, termed recent thymic emigrants (RTEs), are characterized by a naïve phenotype and are essential for the quantitative recovery of T cell counts and diversification of T cell receptor (TCR) repertoire.

Among the cytokines involved in thymic development of T cells, IL-7 and SCF have been identified to play a critical role. IL-7 is a γ-common chain cytokine that is secreted by stromal cells from multiple organs including thymus and it is important for early T cell development [10, 11]. In murine models, exogenous administration of IL-7 after transplantation has been shown to enhance thymopoiesis [12–15]. However, a beneficial effect of exogenous IL-7 administration on thymic dependent T cell regeneration has not been shown in early clinical trials in humans [16, 17]. Furthermore, in addition to thymopoiesis, IL-7 has a much broader role in T cell homeostasis and also affects the thymic-independent pathway of T cell regeneration after lymphodepletion [7, 14, 18]. SCF is a cytokine that plays an important role in hematopoiesis and lymphopoiesis [19]. SCF is produced by non-lymphoid cells, including thymic stromal cells, and its receptor c-kit is highly expressed by the early thymic progenitors [20, 21]. The SCF/c-kit axis is essential in the earliest stages of thymopoeisis [20, 22–24]. In a murine model of transplantation, it has been shown that SCF administration improves post-transplant thymopoiesis largely by promoting intrathymic T cell development, rather than by enhancing entry of prethymically expanded lymphoid progenitors [25]. A synergistic effect of SCF with IL-7 in the improvement of murine thymopoiesis has also been reported [26].

We have previously shown that recovery of thymopoiesis plays a critical role in the attainment of virus-specific immunity and overall survival (OS) in a cohort of adult patients who received double unit UCBT (dUCBT) [27]. Similarly, other groups have shown a beneficial effect of thymic recovery on long term clinical outcomes after transplantation, such as decreased leukemia relapse, risk of opportunistic infections, improved progression free survival (PFS) and overall survival (OS), both after UCBT or HSCT from adult donors [28–31]. The kinetics and clinical significance of circulating levels of IL-7 and SCF in adult UCBT recipients have not been examined. In the present study we sought to investigate the dynamic features and clinical consequences of the thymus-dependent pathway of T cell regeneration after UCBT by analyzing the kinetics of the two major thymotropic cytokines IL-7 and SCF in patient blood, their association with thymic output and quantitative reconstitution of T cell subsets, as well as their prognostic value for clinical outcomes after dUCBT in adult recipients.

## Materials and Methods

### Patients and UCB units

All evaluable patients enrolled in three different phase I/II trials of dUCBT between October 2005 and August 2011 at the Dana Farber/Harvard Cancer Clinical Center were included in the present study [32–34]. All trial protocols were approved by the Institutional Review Board of the Dana Farber/Harvard Cancer Center and written informed consent was obtained from all patients prior to enrollment. The trials were prospectively registered at http://www.clinicaltrials.gov (NCT00133367, NCT00393380 and NCT00890500). The eligibility criteria, detailed design and clinical outcomes of each trial have been previously reported. In brief, patients were eligible for enrollment if they were adults, had a hematologic malignancy and lacked a suitable related (6/6 or 5/6 HLA-A, HLA-B, and HLA-DRB1 matched) or unrelated (10/10 HLA-A, HLA-B, HLA-C, HLA-DRB1, and HLA-DQ matched) donor. UCB units were obtained from national and international cord blood banks. Each individual UCB unit was required to have a minimum of 1.5 × 10^7^ total nucleated cells (TNC)/kg prior to cryopreservation, and the two UCB units selected for each subject were required to provide a minimum of combined pre-cryopreservation cell dose of 3.7 × 10^7^ TNC/kg. UCB units were required to be a 4/6 match or better at the allele level for HLA-A,-B, and-DRβ1 with each other and with the recipient. The UCB units were hierarchically selected on the basis of higher cell dose, greater HLA match and younger age of the unit.

### Treatment protocol

Fifty one patients received reduced intensity conditioning (RIC) regimen with fludarabine (30mg/m^2^/day) for 6 consecutive days (days -8 through -3; total dose 180mg/m^2^), melphalan (100mg/m^2^) on day -2 only, and rabbit antithymocyte globulin (Thymoglobulin, 1–1.5 mg/kg/day) on days -7, -5, -3, -1. Nine patients received a myeloablative conditioning (MAC) regimen of cyclophosphamide 1800 mg/m2/day on days -5 and -4 (total dose 3600 mg/m2), fludarabine 25 mg/m2/day on days -6, -5, -4 (total dose 75 mg/m2) and total body irradiation (TBI) 1400 cGy in 7 divided fractions (days -3, -2, -1, and 0). In addition, the 9 MAC recipients and 4 RIC recipients received commercial human PTH 1–34 (teriparatide) 100 mg/day given as 5 s.c. injections on days 1–28 of the study or until neutrophil engraftment on a research protocol. The two UCB units were infused sequentially between one and six hours apart on day 0. Thirty nine patients received two unmanipulated UCB grafts, whereas 21 patients received one unmanipulated and one ex vivo dimethyl-prostaglandin E2 (dmPGE2) treated UCT unit on another research study protocol. Following transplantation, patients received transfusion support and Filgastrim (5 mcg/kg/day) was administered from day +5 until an absolute neutrophil count higher than 2.0 × 10^9^ cells/L for two consecutive days. Graft vs. host disease (GvHD) prophylaxis began on day -3 and consisted of tacrolimus (0.05 mg/kg for a target serum level of 5–10 ng/mL), in combination with sirolimus or mycophenolate mofetil (MMF). GvHD prophylaxis agents were tapered from day 100 through 180 for patients with no evidence of GvHD.

### Immunophenotyping

Patient blood samples were collected before administration of conditioning chemotherapy and at 4 weeks, 8 weeks, 100 days, 6 months and 1 year after transplantation. Peripheral blood mononuclear cells (PBMCs) were isolated using Ficoll-Paque Plus (GE Healthcare) and stained with fluorescence-conjugated monoclonal antibodies for lineage specific marker analysis, using a BD FACSCanto flow cytometer (BD Biosciences)

### Cytokine measurements

Patient samples were collected before transplantation and at 4 weeks, 8 weeks, 100 days, 6 months and 1 year after transplantation. Plasma was prepared by centrifugation of blood and stored at -80°C. For each time point, the cytokines Interleukin 7 (IL-7) and Stem Cell Factor (SCF) were measured with commercial Colorimetric Sandwich ELISA kits (R&D Systems), according to manufacturer’s instructions.

### TREC analysis

TREC analysis was performed according to a previously described protocol [35]. DNA was isolated from PBMCs with the use of the QIAmp DNA Mini Kit (QIAGEN). Quantitation of signal-joint TCR excision circle (sjTREC) DNA was performed by Quantitative-Competitive Polymerase Chain Reaction (QC-PCR), using Rotor-Gene 6000 thermal cycler (Corbett Life Science). The standard curve was prepared with 10-fold dilutions of a plasmid containing the sjTREC sequence (kindly provided by Dr Daniel Douek, National Institute of Allergy and Infectious Diseases).

### Statistical analysis

The Wilcoxon rank-sum test was used to test differences in continuous variables between groups. Correlation between continuous variables was assessed using Spearman rank test. Overall survival (OS) was defined as the time from transplantation to death from any cause, whereas progression-free survival (PFS) was defined as the time from transplantation to progression or death from any cause. OS and PFS were estimated using the Kaplan-Meier method. Cumulative incidence of GVHD, relapse and non-relapse mortality (NRM) were calculated in the competing risks framework considering relapse/death without developing GVHD, NRM, and relapse as competing events, respectively [36, 37]. Multivariable and univariable Cox regression analysis was performed for OS, PFS, NRM, and relapse treating each of TREC, IL-7, SCF as a time-dependent variable. Due to the limited number of events, in each model for OS, PFS, NRM, and relapse, univariable analysis was initially performed for variables listed in Table 1; variables with p-value less than 0.1 were included in the models. These variables are age, male patient with female donor, disease (AML or MDS vs other) along with TREC, IL-7 or SCF. For chronic GvHD (cGVHD), due to the small number of events, only univariable Cox model was performed. In addition, for the difference in IL-7 and SCF levels from the baseline to each time point after transplantation, pairwise comparison was made using Wilcoxon-signed-rank test and then repeated measures analysis was explored after adjusting for aforementioned variables with an appropriate covariance structure for each parameter of interest. Prior to performing regression analysis, TREC, IL-7 and SCF were log10 or natural log transformed, as appropriate, to meet the normality assumption. All *P* values were based on 2-sided tests. The significance level was set to 0.01 to adjust for multiplicity in differences and correlations of TREC, IL-7 and SCF, but set to 0.05 for association with clinical outcome. All calculations were done using SAS 9.3 (SAS Institute Inc, Cary, NC), and R version 2.13.2 (the CRAN project).

**Table 1:** Baseline characteristics of 60 dUCBT patients and cord blood units.

	N	%
Total	60	100
Age, median (range)	47 (19, 67)	
Patient Sex		
Male	33	55
Female	27	45
Male patient & Female donor	25	41.7
Diagnosis		
AML	21	35
ALL	4	6.7
CLL/SLL/PLL	3	5
CML	1	1.7
MDS	9	15
MPD	1	1.7
Hodgkin Disease	6	10
NHL	12	20
Anemia/ Red Cell Disorder	2	3.3
Other Acute Leukemia	1	1.7
HLA allele match at A, B, DRB1		
4/6+≥4/6	52	86.7
5/6+5/6	5	8.3
Other	3	5.0
Disease Status at conditioning		
in remission	39	65.0
active	19	31.7
missing	2	3.3
Conditioning Intensity		
Fludarabine/Cyclophosphamide/TBI (MAC)	9	15
Fludarabine/Melphalan/ATG (RIC)	51	85
GVHD prophylaxis		
Tac/MMF	12	20
Tac/Sir	48	80
Total TNC infused (x10^8^ cells/kg)		
median (range)	0.42 (0.21, 0.68)	
Total CD34+ infused (x10^6^ cells/kg)		
median (range)	0.20 (0.03, 1.15)	

## Results

### Patient characteristics and clinical outcomes

Sixty patients with high risk hematologic malignancies were included in the present study (Table 1). The median recipient age was 47 (19–67) years. The most common indication for transplantation was AML/MDS in 50% of patients, followed by lymphoid malignancies. The majority of patients (85%) received RIC regimen consisting of fludarabine/melphalan/ATG whereas 15% of patients received MAC regimen with fludarabine/cyclophosphamide/TBI. The infusion of two UCB grafts provided a median TNC dose per patient of 4.2x10^7^ cells/kg, and a median CD34+ cell dose of 2x10^5^ cells/kg.

The median follow up among survivors was 70 (32–101) months (Table 2). Grade II-IV acute GvHD (aGvHD) occurred in 7 patients by Day+100 and in 8 patients (13%) by Day+180. Chronic GvHD developed in 10 (17%) and 15 (25%) patients by 1 and 2 years respectively. The 5-year cumulative incidence of NRM was 34%. During the study follow-up period, 25 patients (41%) relapsed. The 5-year OS and PFS were 39% and 25%, respectively.

**Table 2:** Clinical outcomes of dUCBT recipients.

Outcome	% (95% CI)
Cumulative Incidence of Grade II-IV aGVHD	
at D100	12 (5.1, 21)
at D180	13 (6.2, 23)
Cumulative Incidence of cGVHD	
1-yr	17 (8.5, 27)
2-yr	25 (15, 37)
5-yr Cumulative Incidence of	
NRM	34 (22, 46)
Relapse	41 (28, 53)
5-yr PFS	25 (15, 36)
5-yr OS	39 (26, 51)

Median f/u time among survivors: 70 months (range 32, 101)

### Kinetics of IL-7 and SCF after dUCBT

**Fig 1:**
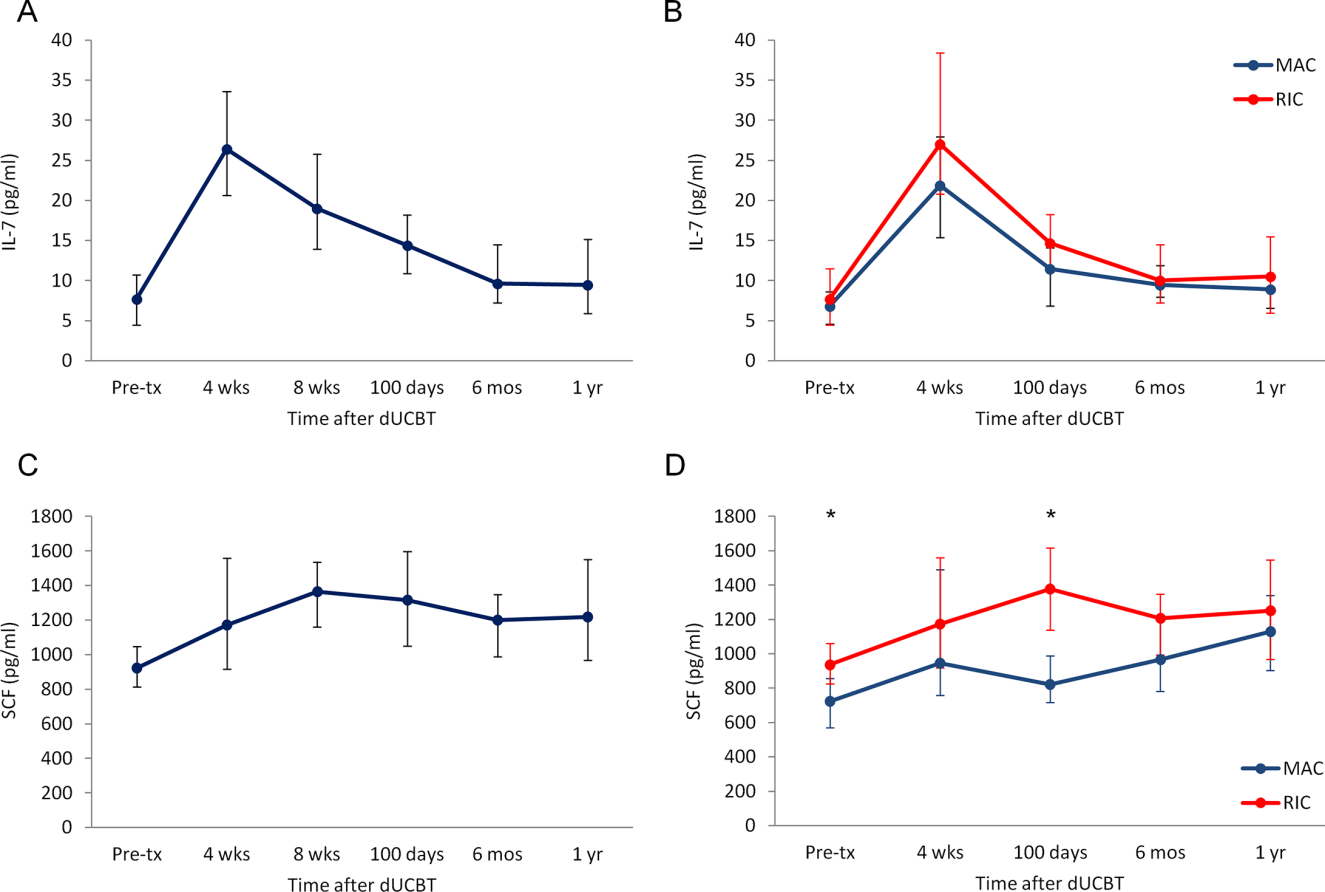
Kinetics of plasma IL-7 and SCF levels after dUCBT. **(A)** Median IL-7 levels from baseline through 12 months post-transplantation. **(B)** Median IL-7 levels of patients who received RIC regimen and median IL-7 levels of patients who received MAC regimen were plotted separately. **(C)** Median SCF levels from baseline through 12 months post-transplantation. **(D)** Median SCF levels of patients who received RIC regimen and median SCF levels of patients who received MAC regimen were plotted separately. Error bars denote 25^th^ and 75^th^ percentiles. (* p<0.05)

Median IL-7 levels rose by more than 3-fold from 7.7 pg/mL before conditioning to a peak of 26.4 pg/mL at 4 weeks. Plasma levels of IL-7 remained significantly elevated through 100 days post dUCBT (p<0.0001), and subsequently declined and reached near pre-transplant levels by 1 year (Fig 1A). There were no statistically significant differences in the median values and kinetics of IL-7 between patients who received RIC vs. MAC regimens (Fig 1B). Plasma SCF showed a less than 2-fold increase from baseline, reaching peak levels at 8 weeks after dUCBT. Notably, plasma levels of SCF were steadily increased and remained significantly higher than pretransplant levels (p<0.0001) at all time points during the first year after dUCBT (Fig 1C). Compared with RIC recipients, patients receiving MAC regimen had lower levels of SCF at baseline (p = 0.01) and at 100 days after dUCBT (p = 0.01) (Fig 1D). Within the group of RIC recipients, patients who received one unmanipulated and one *ex vivo* dmPGE2-treated UCB graft had no differences in SCF and IL-7 levels at baseline and until 100 days after dUCBT. However, in this patient group, levels of SCF and IL-7 remained elevated through 1 year after transplantation (S1 Fig). The mechanisms and implications of this finding are currently under investigation in an ongoing phase II clinical trial but do not affect the results and conclusions of the present study. IL-7 and SCF levels were positively correlated at all time points after dUCBT, with the exception of their preconditioning values (S1 Table).

### Plasma levels of IL-7 and SCF inversely correlate with T cell subsets after dUCBT

In a subset of patients (n = 27), in whom we had sufficient material to generate a complete series of immune reconstitution data, we examined whether the levels of IL-7 and SCF might display any correlation with the quantitative recovery of T cell subsets after dUCBT. All patients in this subset received two unmanipulated grafts and were treated with identical RIC regimen and the same immunosuppression for GvHD prophylaxis after transplantation [32]. The timeline of quantitative T cell recovery in this subgroup has previously been reported [27]. We observed an inverse correlation between IL-7 levels and CD4^+^ counts throughout the first year after dUCBT (Table 3). At each time point, the CD4^+^CD45R0^+^ memory subset showed a stronger inverse correlation with IL-7 levels as compared to the naive CD4^+^CD45RA^+^ subset, with the exception of the 4 week time point. CD8^+^ counts also inversely correlated with IL-7 levels after dUCBT although the inverse correlation is not as strong as in CD4^+^ (Table 3). Treg lymphocytes, defined as CD4^+^CD25^+^ cells, showed an inverse correlation with IL-7 levels overall. In contrast to IL-7, we did not observe a significant correlation between SCF levels and T cell subsets for most time points after dUCBT. However, at 1 year after transplantation, SCF levels showed a statistically significant inverse correlation with CD4^+^ counts and specifically the naïve CD4^+^CD45RA^+^ subset of T cells (Table 4).

**Table 3:** Correlation of IL-7 levels with T cell subsets during the first year after dUCBT.

IL-7		4 weeks	8 weeks	100 days	6 months	1 year
CD4^+^	r	-0.42	-0.51	-0.63	-0.60	-0.51
	p-value	0.10	0.02	0.003	0.007	0.08
CD4^+^CD45RA^+^	r	-0.54	-0.43	-0.41	-0.33	-0.30
	p-value	0.04	0.06	0.07	0.16	0.33
CD4^+^CD45R0^+^	r	-0.29	-0.46	-0.67	-0.67	-0.49
	p-value	0.25	0.047	0.001	0.002	0.09
CD4^+^CD25^+^	r	-0.32	-0.34	-0.21	-0.54	-0.27
	p-value	0.21	0.15	0.38	0.02	0.39
CD8^+^	r	-0.37	-0.49	-0.48	-0.29	-0.34
	p-value	0.14	0.03	0.03	0.23	0.26
CD8^+^CD45RA^+^	r	-0.56	-0.50	-0.38	-0.10	-0.32
	p-value	0.02	0.03	0.10	0.68	0.29
CD8^+^CD45R0^+^	r	-0.35	-0.46	-0.51	-0.53	-0.20
	p-value	0.17	0.045	0.02	0.02	0.52

r denotes correlation coefficient from Spearman rank test

**Table 4:** Correlation of SCF levels with T cell subsets during the first year after dUCBT.

SCF		4 weeks	8 weeks	100 days	6 months	1 year
CD4^+^	r	-0.17	-0.41	-0.12	-0.23	-0.78
	p-value	0.52	0.07	0.61	0.34	0.002
CD4^+^CD45RA^+^	r	-0.33	0.01	0.09	-0.30	-0.79
	p-value	0.23	0.97	0.71	0.21	0.001
CD4^+^CD45R0^+^	r	-0.02	-0.47	-0.13	-0.25	-0.65
	p-value	0.94	0.04	0.57	0.30	0.02
CD4^+^CD25^+^	r	-0.29	-0.23	0.32	-0.02	-0.60
	p-value	0.26	0.35	0.17	0.95	0.04
CD8^+^	r	-0.07	-0.50	-0.05	-0.09	-0.20
	p-value	0.79	0.02	0.84	0.7	0.52
CD8^+^CD45RA^+^	r	-0.04	-0.42	0.10	-0.12	-0.24
	p-value	0.89	0.08	0.68	0.64	0.42
CD8^+^CD45R0^+^	r	-0.08	-0.49	-0.07	-0.18	-0.12
	p-value	0.76	0.03	0.77	0.47	0.69

r denotes correlation coefficient from Spearman rank test

### Kinetics of TRECs after dUCBT and relationship to serum levels of IL-7 and SCF

**Fig 2:**
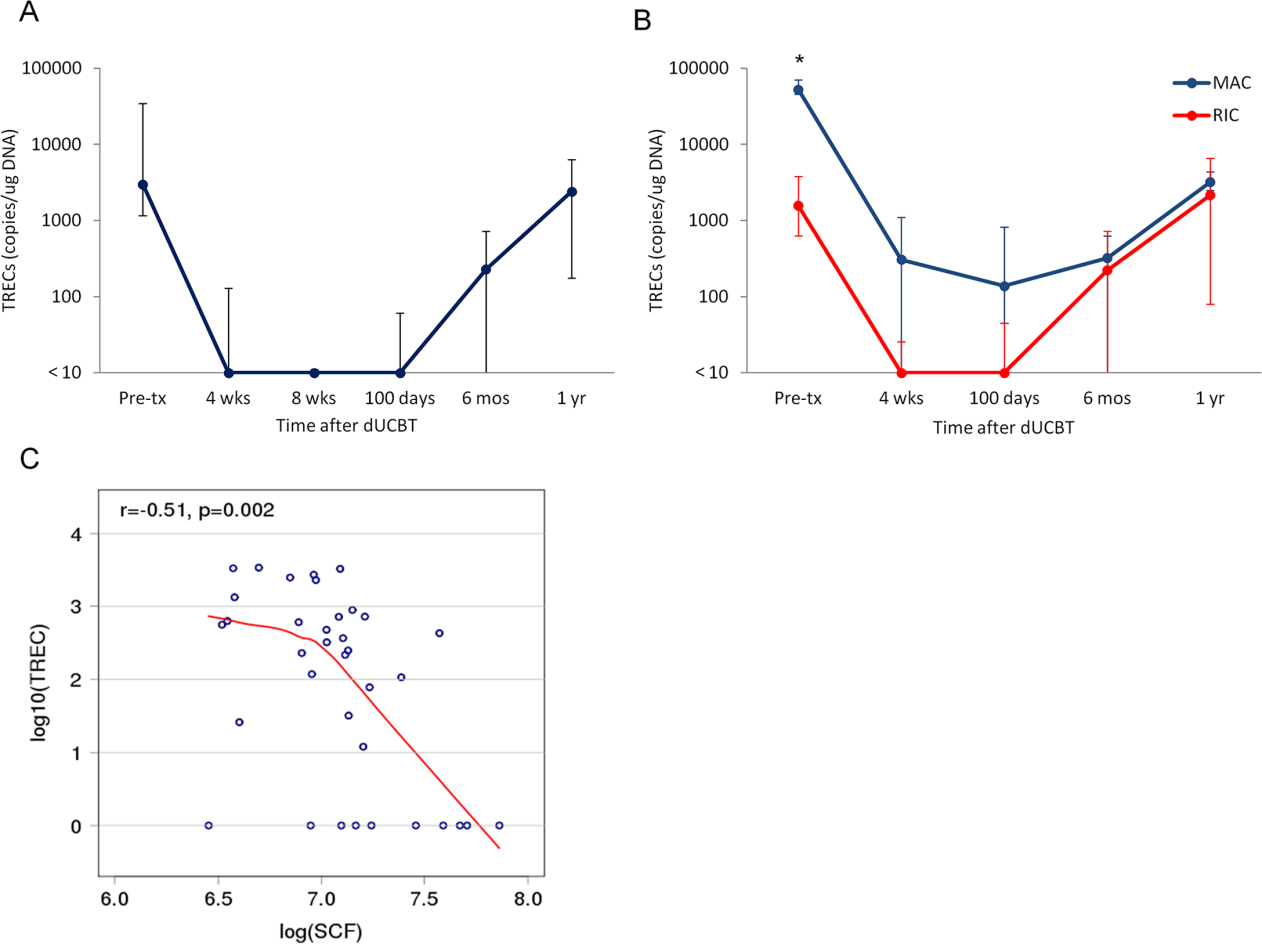
Reconstitution of TRECs after dUCBT. **(A)** Median TREC values at various time points after dUCBT **(B)** Median TREC levels of patients who received RIC regimen and median TREC levels of patients who received MAC regimen were plotted separately. The limit of detection for the assay is 10 copies/ug DNA. Error bars denote 25^th^ and 75^th^ percentiles **(C)** TREC values inversely correlate with plasma SCF levels at 6 months after dUCBT. Log10TREC vs. logSCF at 6 months after dUCBT are shown. (* p<0.05)

TRECs are circular molecules of episomal DNA that represent byproducts of the T cell receptor gene rearrangement events that take place during the intrathymic T cell development [35]. TRECs can be measured with quantitative competitive PCR (QC-PCR) and have been used as a surrogate marker of thymopoiesis. Before transplantation, all but one evaluable patient had detectable TRECs with a median value of 2981 copies/ug DNA (Fig 2A). There was a wide interpatient variability in TREC levels at baseline. Patients receiving MAC conditioning had significantly higher baseline levels compared to RIC recipients (p = 0.0007), likely reflecting the younger age of patients who were eligible for MAC protocol. No significant differences were observed at subsequent time points (Fig 2B). TREC levels remained either undetectable or extremely suppressed through 100 days. By 6 months, 26 of 37 evaluable patients (70%) had detectable TREC levels, albeit the median TREC value (229 copies/ug DNA) remained below the normal range. At 1 year after dUCBT, 27 of 32 evaluable patients (84%) had recovered thymopoiesis, with a median TREC value (2405 copies/ug DNA) within low normal limits [38]. The increase in TREC levels starting at 6 months after dUCBT was accompanied by a parallel increase in the T cell counts [27] and was preceded by a decline in IL-7 and SCF levels (Figs 1A, 1C and 2A). At 6 months after UCBT, we observed a statistically significant inverse correlation between TRECs and SCF levels (r = -0.51, p = 0.002) (Fig 2C). An inverse correlation was also observed at 1 year, although it did not reach statistical significance (r = -0.35, p = 0.06). In contrast, we did not detect a significant correlation between IL-7 and TREC levels at any timepoint after dUCBT, suggesting that SCF but not IL-7 rather has a direct effect on mechanisms related to the regeneration of thymic function and lymphocyte output.

### Prognostic value of IL-7, SCF and TREC levels on clinical outcomes after dUCBT

Our data suggest that high IL-7 and SCF levels are associated with delayed T cell subset and thymic reconstitution after UCBT. We and others have shown that early lymphocyte count and thymic recovery are favorable prognostic factors for survival after UCBT or conventional HSCT [9, 27, 29, 39]. Therefore, we next examined whether TRECs, IL-7 and SCF levels might have a distinct prognostic value for long term outcomes after UCBT, specifically OS, PFS, NRM and relapse. In multivariable analysis, higher TREC levels were associated with lower NRM (p = 0.006), improved PFS (p = 0.0498) and OS (p = 0.02), (Table 5). In contrast, high levels of IL-7 and SCF were associated with increased risk of death (p = 0.002 and p = 0.001, respectively) and higher NRM (p = 0.03 and p = 0.01, respectively). Neither TREC nor cytokine levels were found to have an effect on relapse risk. Finally, higher IL-7 was associated with increased risk of cGVHD (p = 0.03) (Table 5).

**Table 5:** Multivariable analysis for OS, PFS, NRM, relapse and cGvHD.*

Outcome		HR	95% CI		p-value
**OS**	Log10 (TREC)	0.72	0.54	0.96	0.02
	Log(IL-7)	2.80	1.47	5.32	0.002
	Log (SCF)	2.98	1.62	5.50	0.001
**PFS**	Log10 (TREC)	0.78	0.60	1.00	0.0498
	Log(IL-7)	1.16	0.68	1.98	0.59
	Log (SCF)	1.37	0.76	2.47	0.29
**NRM**	Log10 (TREC)	0.59	0.40	0.86	0.006
	Log(IL-7)	2.51	1.08	5.83	0.03
	Log (SCF)	3.00	1.28	7.01	0.01
**Relapse**	Log10 (TREC)	1.00	0.68	1.47	1.00
	Log(IL-7)	0.73	0.37	1.47	0.38
	Log (SCF)	0.77	0.31	1.94	0.58
cGVHD*	Log10 (TREC)	0.85	0.54	1.34	0.48
	Log(IL-7)	2.67	1.09	6.55	0.03
	Log (SCF)	0.60	0.14	2.54	0.49

OS, PFS, NRM & relapse are from multivariable Cox models adjusting patient age, male patient with female donor and disease (AML/MDS vs other).

*cGVHD is from a univariable Cox model.

## Discussion

In the present study we examined the kinetics of IL-7 and SCF in relation to quantitative T cell and thymic reconstitution in adult patients undergoing dUCBT, as well as their prognostic value on clinical outcomes of dUCBT. We observed that plasma levels of IL-7 and SCF increase and remain elevated for several months after dUCBT, and inversely correlate with the numbers of specific T cell subsets at certain time points after transplantation. Notably, SCF but not IL-7 inversely correlated with TREC levels, indicating a selective role of this cytokine in the regeneration of thymic output after dUCBT. Furthermore, our findings suggest that elevated levels of IL-7 and SCF are associated with lower OS and higher NRM, and elevated IL-7 levels are also associated with cGvHD.

The IL-7 receptor, CD127, is expressed in nearly all circulating T lymphocytes but highest levels are present in naïve lymphocytes and lymphocyte progenitors [40]. It has been long observed that circulating levels of IL-7 inversely correlate with T cell counts [41]. In the setting of lymphopenia, the number of IL-7 receptor-bearing lymphocytes is decreased resulting in elevated plasma levels of IL-7, likely due to decreased consumption, a finding that emphasizes the role of IL-7 as a key regulator of T cell homeostasis [40–42]. This observation has been replicated in several recent studies examining the kinetics of IL-7 after HSCT [43–46]. In the present study we demonstrated a protracted elevation of IL-7 levels after dUCBT in adults. This finding is in contrast to previous observations after MAC or RIC HSCT from adult donors [43–47]. Although the inclusion of ATG in the RIC recipients of our study might be a contributing factor [46], we observed a protracted elevation of IL-7 regardless of the conditioning regimen. We hypothesize that this finding might be due to the low number of HSC or T cells contained in UCB grafts and the delayed recovery of lymphocyte counts associated with UCBT. Consistent with this hypothesis, we observed that circulating IL-7 levels inversely correlated with various T cell subsets at several time points after dUCBT. This relationship was more pronounced with CD4^+^ counts, in accord with previous observations suggesting that IL-7 levels may be regulated primarily by CD4^+^ T cell mass [7, 42].

In contrast to IL-7, a relatively small fluctuation in plasma SCF after chemotherapy and autologous transplantation has been reported previously [48]. However, data in allogeneic transplantation from adult HSC donors or UCB are lacking. We observed a modest increase in SCF levels after dUCBT and median values remained steadily increased through 1 year. Compared with RIC recipients, MAC recipients had significantly lower SCF levels at baseline and at 100 days. At the same time points, MAC recipients also had higher TRECs, although this difference did not reach statistical significance at the day 100 time point. While the higher pre-transplant TREC levels in MAC vs. RIC recipients are likely related to the younger age of patients eligible for MAC protocol, the use of ATG in RIC recipients might have been an additional contributing factor to the trend for lower TREC levels at 100 days in these patients [29], We observed a statistically significant inverse correlation between SCF and TREC levels at 6 months and a trend for inverse correlation at 1 year after dUCBT. Notably, at 1 year there was also a strong inverse correlation between SCF and the naïve CD4^+^CD45RA^+^ subset. Although it is unclear if circulating SCF levels reflect the concentrations of SCF in the microenvironment of the thymus, these observations support a pivotal role of this cytokine in the thymus-dependent pathway of immune reconstitution after HSCT. The inverse correlation between SCF and TREC levels is likely due to increased consumption because the SCF receptor, c-kit, is highly expressed by the early thymic progenitors [20, 21], and is consistent with the essential role of the SCF/c-kit axis in the earliest stages of thymopoiesis [20, 22, 23]. In contrast, we did not find a significant correlation between plasma IL-7 and TREC levels. Therefore, IL-7 may preferentially mediate the homeostatic expansion of post-thymic T cells in the periphery, and may play a lesser role in the thymic-dependent pathway of immune reconstitution [49, 50].

In murine models of transplantation, it has been shown that IL-7 plays a critical role in the pathogenesis of GvHD [51–53]. The elevated levels of IL-7 in the early post transplant period may lead to the expansion of alloreactive donor T cells infused with the graft and predispose to the development of GvHD [54]. Indeed, high IL-7 levels in the early post-transplant period correlate with increased incidence and severity of aGvHD in recipients of HSCT from adult donors [43–46]. Similarly, in our study, patients who developed grade II-IV aGvHD had significantly higher IL-7 levels at 1 month (data not shown). Additionally, we observed that patients who had persistently elevated IL-7 levels had higher incidence of cGvHD. This finding contradicts two previous reports showing no association between early IL-7 levels and cGvHD after HSCT [43, 45]. It is possible that, in these previous studies, IL-7 levels were not followed long enough to allow detection of association with cGvHD. Alternatively, our findings may be related to the unique features of cGvHD in UCBT, which often presents with characteristics of aGvHD and less frequently of classical cGvHD [55–57]. The correlation of IL-7 levels with both GvHD and poor quantitative T cell recovery can be explained by the well established fact that the thymus is a sensitive target of both aGvHD and cGvHD [9, 58]. Furthermore, the use of immunosuppressive agents to control GvHD may compromise the process of immune reconstitution.

Previous studies have shown that thymic reconstitution is associated with favorable outcomes after UCBT or HSCT [27–29, 31]. In the present study, we observed detectable thymic activity by TREC assay in 70% at 6 months and 84% of subjects at 1 year, with return to near pre-transplant levels at 1 year. In multivariable analysis, we observed that higher TREC level was associated with improved OS and PFS and lower NRM, whereas there was no effect on risk of relapse. Because our data showed that IL-7 and SCF inversely correlated with quantitative T cell recovery and SCF levels also inversely correlated with thymic reconstitution, we examined the prognostic value of these two cytokines on long term outcomes of dUCBT. Both IL-7 and SCF levels were adversely associated with OS and higher NRM. One could hypothesize that the higher incidence of GvHD in patients with elevated IL-7 might account for the higher NRM. However, GvHD after UCBT is associated with a relatively low NRM [59] and the development of cGvHD after UCBT has been favorably associated with both OS and PFS, likely due to graft vs. malignancy effect without excessive mortality from GvHD [60]. We did not detect any association of SCF with GvHD and neither cytokine had any correlation with relapse risk. Therefore, our data support that the adverse effect of high IL-7 and SCF on OS and NRM is likely due to higher rates of infectious complications, as a result of prolonged immunodeficiency.

In summary, two main thymotropic cytokines, IL-7 and SCF, show prolonged elevation after dUCBT and are associated with poor quantitative T cell and thymic reconstitution. Persistently elevated levels are adverse prognostic factors for NRM and OS, therefore may identify high risk patients after dUCBT.

## Supporting Information

S1 Fig Kinetics of plasma IL-7 and SCF levels after dUCBT in RIC recipients. **(A)** Median IL-7 levels of patients who received RIC regimen followed by one dmPGE2 treated and one unmanipulated UCB graft (RIC+PGE2-UCBT) vs. median IL-7 levels of patients who received two unmanipulated UCB grafts (RIC+dUCBT) through 12 months post-transplantation. **(B)** Median SCF levels of RIC+PGE2-UCBT vs. RIC+dUCBT recipients through 12 months post-transplantation. Error bars denote 25th and 75th percentiles. (* p<0.05).
(PDF)

S1 Table Correlation of IL-7 and SCF plasma levels during the first year after dUCBT. Levels of IL-7 and SCF were assessed in patients’ plasma at the indicated time points after dUCBT and correlation was analyzed.
(PDF)
