# Supplementary material for: IL-7 and SCF Levels Inversely Correlate with T Cell Reconstitution and Clinical Outcomes after Cord Blood Transplantation in Adults
Source: PLoS One. 2015 Jul 15;10(7):e0132564. doi: 10.1371/journal.pone.0132564 (PMC4503696; doi:10.1371/journal.pone.0132564)
Supplement: S1 Fig — (A) Median IL-7 levels of patients who received RIC regimen followed by one dmPGE2 treated and one unmanipulated UCB graft (RIC+PGE2-UCBT) vs. median IL-7 levels of patients who received two unmanipulated UCB grafts (RIC+dUCBT) through 12 months post-transplantation. (B) Median SCF levels of RIC+PGE2-UCBT vs. RIC+dUCBT recipients through 12 months post-transplantation. Error bars denote 25th and 75th percentiles. (* p<0.05). (PDF) [file pone.0132564.s001.pdf]

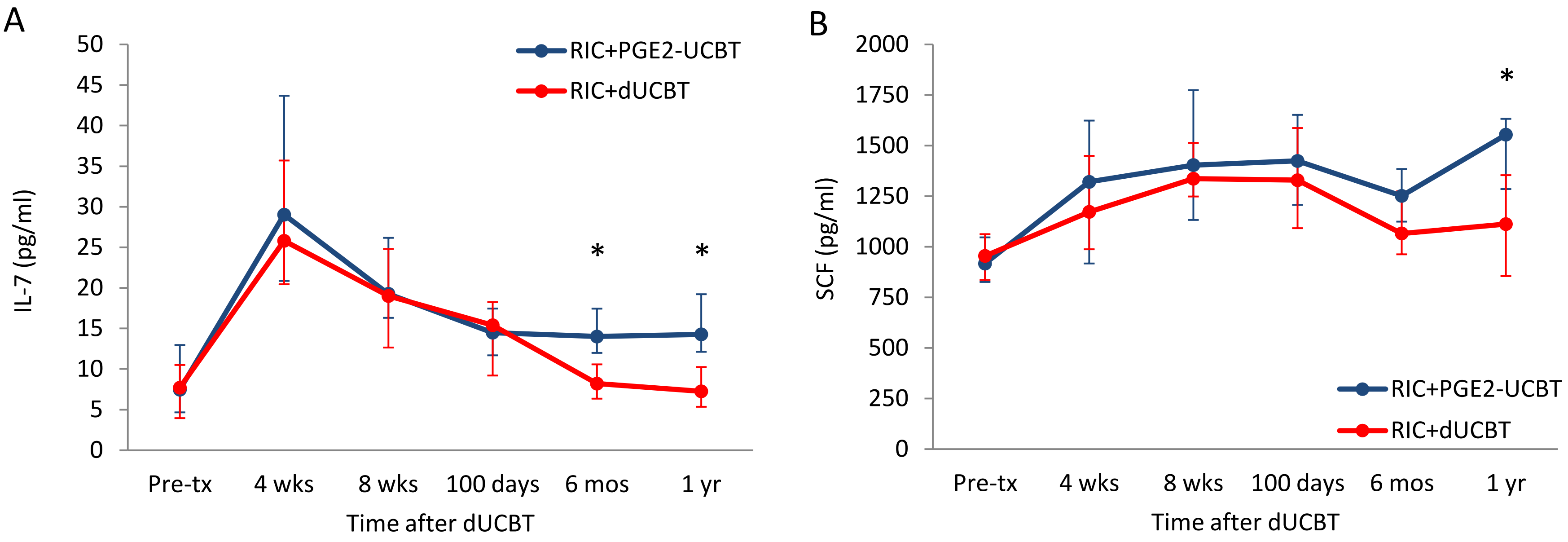

Figure S1. Kinetics of plasma IL-7 and SCF levels after dUCBT in RIC recipients. (A) Median IL-7 levels of patients who received RIC regimen followed by one dmPGE2 treated and one unmanipulated UCB graft (RIC+PGE2-UCBT) vs. median IL-7 levels of patients who received two unmanipulated UCB grafts (RIC+dUCBT) through 12 months post-transplantation. **(B)** Median SCF levels of RIC+PGE2-UCBT vs. RIC+dUCBT recipients through 12 months post-transplantation. Error bars denote 25th and 75th percentiles. (* $p<0.05$)

Figure S1
