# Supplementary material for: IL-7 and SCF Levels Inversely Correlate with T Cell Reconstitution and Clinical Outcomes after Cord Blood Transplantation in Adults
Source: PLoS One. 2015 Jul 15;10(7):e0132564. doi: 10.1371/journal.pone.0132564 (PMC4503696; doi:10.1371/journal.pone.0132564)
Supplement: S1 Table — Levels of IL-7 and SCF were assessed in patients’ plasma at the indicated time points after dUCBT and correlation was analyzed. (PDF) [file pone.0132564.s002.pdf]

Supplementary table 1. Correlation of IL-7 and SCF plasma levels during the first year after dUCBT

Correlation of IL-7 and SCF levels after dUCBT		
Pre-transplant	r<br>p-value	0.05<br>0.70
4 weeks	r<br>p-value	0.54<br><0.0001
8 weeks	r<br>p-value	0.34<br>0.02
100 days	r<br>p-value	0.32<br>0.02
6 months	r<br>p-value	0.37<br>0.02
1 year	r<br>p-value	0.52<br>0.002
r denotes correlation coefficient from Spearman rank test		

Levels of IL-7 and SCF were assessed in patients' plasma at the indicated time points after dUCBT and correlation was analyzed.
